# Development of Real-Time Time Gated Digital (TGD) OFDR Method and Its Performance Verification

**DOI:** 10.3390/s21144865

**Published:** 2021-07-16

**Authors:** Kinzo Kishida, Artur Guzik, Ken’ichi Nishiguchi, Che-Hsien Li, Daiji Azuma, Qingwen Liu, Zuyuan He

**Affiliations:** 1 Neubrex Co., Ltd. 1-1-24 Sakaemachi-dori, Kobe 650-0024, Japan; guzik@neubrex.jp (A.G.); nishiguchi@neubrex.jp (K.N.); li-z@neubrex.jp (C.-H.L.); azuma@neubrex.jp (D.A.); 2 State Key Laboratory of Advanced Optical Communicate Systems and Networks, Shanghai Institute for Advanced Communication and Data Science, Shanghai Jiao Tong University, 800 Dongchuan Rd., Shanghai 200240, China; liuqingwen@sjtu.edu.cn (Q.L.); zuyuanhe@sjtu.edu.cn (Z.H.)

**Keywords:** OFDR type DAS, phase fading solution, high SNR, real-time events detection

## Abstract

Distributed acoustic sensing (DAS) in optical fibers detect dynamic strains or sound waves by measuring the phase or amplitude changes of the scattered light. This contrasts with other distributed (and more conventional) methods, such as distributed temperature (DTS) or strain (DSS), which measure quasi-static physical quantities, such as intensity spectrum of the scattered light. DAS is attracting considerable attention as it complements the conventional distributed measurements. To implement DAS in commercial applications, it is necessary to ensure a sufficiently high signal-noise ratio (SNR) for scattered light detection, suppress its deterioration along the sensing fiber, achieve lower noise floor for weak signals and, moreover, perform high-speed processing within milliseconds (or sometimes even less). In this paper, we present a new, real-time DAS, realized by using the time gated digital-optical frequency domain reflectometry (TGD-OFDR) method, in which the chirp pulse is divided into overlapping bands and assembled after digital decoding. The developed prototype NBX-S4000 generates a chirp signal with a pulse duration of 2 μs and uses a frequency sweep of 100 MHz at a repeating frequency of up to 5 kHz. It allows one to detect sound waves at an 80 km fiber distance range with spatial resolution better than a theoretically calculated value of 2.8 m in real time. The developed prototype was tested in the field in various applications, from earthquake detection and submarine cable sensing to oil and gas industry applications. All obtained results confirmed effectiveness of the method and performance, surpassing, in conventional SM fiber, other commercially available interrogators.

## 1. Introduction

More than a decade ago, distributed acoustic sensing (DAS) became a popular measurement method, especially in the oil and gas industry [1]. In principle, the phase change between an incident light pulse and the returned Rayleigh scattered lights [1] is measured and determined, and using those data to detect and locate acoustic events. Typical applications of DAS include also seismic profiling, hydraulic fracturing monitoring, and intrusion detection. Those applications and corresponding technical requirements, which need to be met, are listed in Table 1 [2]. The first of the applications listed there, seismic wave measurements, has almost no real time requirements regarding processing time. In the other two applications, the size of the datum is in the order of terabytes per day, but it contains only a small amount of the necessary information. The necessary information is required to be output in real time.

Almost all DAS implementations, so far, are based on the optical time domain reflectometry (OTDR) technique [1]. Moreover, some of the authors of this paper had developed an OTDR-type interrogator, which used phase polarization diversity [3] to improve signal quality and reduce noise. The OTDR method has inherent problems, such as low sensitivity of acoustic measurements, Rayleigh scattering phase fading phenomena, and noise components of the system in the frequency domain contaminating the results. Despite that, some excellent results have been published by the industry; in each case, special methods were developed to deal with those OTDR related problems.

The frequency counterpart of OTDR, the optical frequency domain reflectometry (OFDR) type methods were also formulated. For those, time-gated digital (TGD)-OFDR, which applies a time gate to the chirp signal, has been studied [4,5]. The TGD method, when used for digital analysis, demonstrated excellent performance in the laboratory [4]. This paper describes the effort undertaken to develop a commercial level interrogator, as well as verification of its performance in real, in-the-field applications. It also contains and discusses obtained results and compares them with other works.

The TGD-DAS technology uses long optical pulses to solve the signal SNR problem. Chirp’s pulse compression method can achieve a high spatial resolution of about 100 times. We also solved the phase-fading problem by dividing the frequency band of the chirp. Thus, TGD-DAS is expected to be the most promising DAS technology. During the presentation of the sensing method, SEAFOM [6] naming and technical terms are used.

## 2. System Configuration

The first (proof-of-concept measurements reported in [7]) were made on a laboratory system with a single data channel, capable of acquiring signals for the maximum duration of only a few seconds.

**Figure 1:**
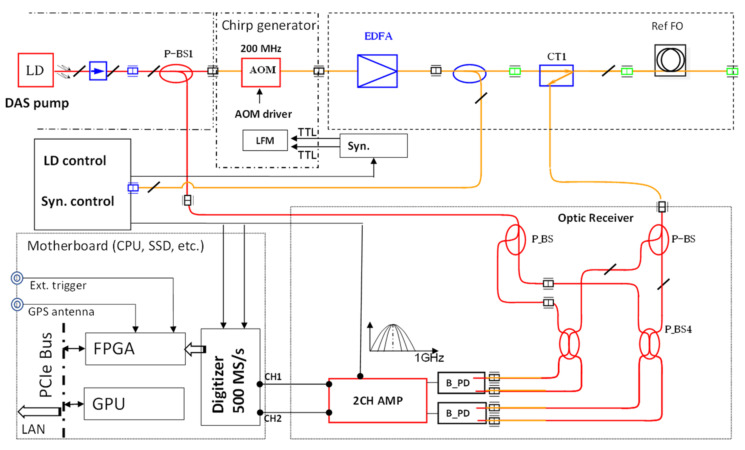
Configuration of prototype DAS NBX-S4000.

During the development described in this paper, we designed the system to meet three targets: being deployable in the field (commercial operational level), being capable of real-time data processing and, finally, and most importantly, to obtain signal in standard single-mode (SM) fibers of quality matching, or surpassing that with modified (referred to as engineered) fibers. The result of the system development is presented in Figure 1, which shows the configuration of the DAS prototype NBX-S4000 using TGD-OFDR.

As the light source, the fiber laser with a narrow linewidth (~100 Hz) is used. The chirp waveform is generated using the Acousto-Optic Modulator (AOM) of 200 MHz with the resulting chirp frequency range 145 MHz to 245 MHz. The signal is then acquired on a polarization diversity component and analyzed separately for both polarization components, P and S. Please note that the polarization components are denoted here by convention used in optics, and are not related to the P- and S-waves as used in seismology and other applications.

**Figure 2:**
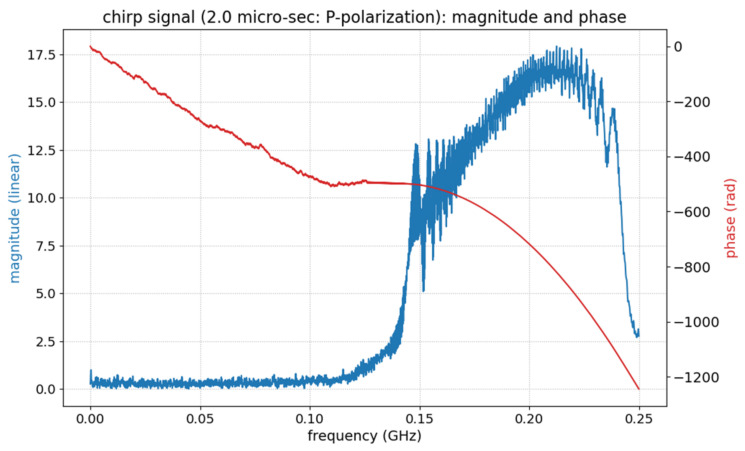
Chirp signal (P-polarization component) in NBX-S4000.

An example of a chirp signal (specifically its P-polarization component), as recorded in the NBX-S4000 instrument, is presented in Figure 2. We should note that this signal shown in this figure is obtained by connecting to the output fiber in the heterodyne receiver in the system. As a result, due to local reference light, the high frequency of amplitude is observed.

Once the signal is acquired and digitized, the signal processing is performed. The algorithm for signal processing is the same as already presented in [4,5], so it will not be repeated here. The resulting reflection coefficient trace is complex, and the magnitude part represents the reflection intensity trace of the measured fiber.

**Figure 3:**
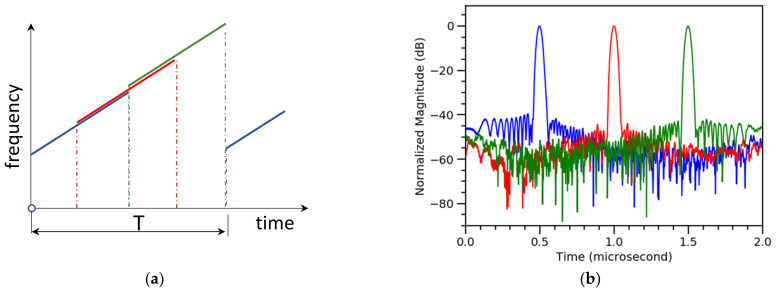
Signal decoding scheme. (**a**) Chirped pulse and sub-division into bands (offset for visibility, frequency change is linear over entire range); (**b**) corresponding frequency bands used during signal processing.

Three signal bands, using three decoding filters for each polarization component, are extracted from the acquired signal. This is schematically presented in Figure 3a. In this figure, the band frequencies are offset for the sake of readability, to indicate that they overlap. The normalized magnitudes of the bands in dB are shown in Figure 3b, where each band is indicated by a corresponding (matching) color.

The decoding step, described above, is performed on an integrated, field-programmable gate array (FPGA). When used with a specially developed and optimized processing algorithm, FPGA makes it possible to obtain results in real-time, despite processing six data components (three bands for each polarization).

In many DAS systems, the actual complexity of the signal processing depends mainly on the part handling the occurrences of the phenomena referred to as phase fading and signal-to-noise ratio (SNR), in general. The achieved signal quality on the developed interrogator unit is examined in detail in the next section.

## 3. Signal-to-Noise Ratio (SNR)

The phase shift of the backscattered light is linearly proportional to the vibration/acoustic amplitude. However, along the fiber there exist (many) randomly distributed locations, where the intensity of backscattering is extremely low. This effect is mainly caused by interference fading or by polarization fading [3,4].

As the SNR is low in general, the extracted phase at these fading points contain large noise, making estimation and actual phase change calculations extremely difficult. An efficient method to reduce the fading noise is to vary the frequency of the probe light and average independent Rayleigh backscattering traces. In TGD-DAS, this is achieved by dividing the (chirp) signal into (overlapping) bands. Each band is then individually processed.

**Figure 4:**
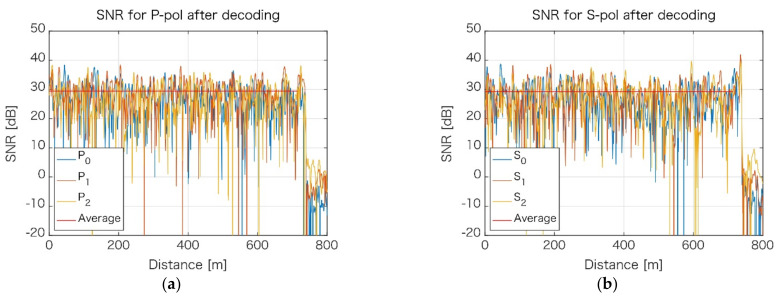
Signal-to-noise distribution for individual frequency bands. (**a**) P-polarization component; (**b**) S-polarization component.

**Figure 5:**
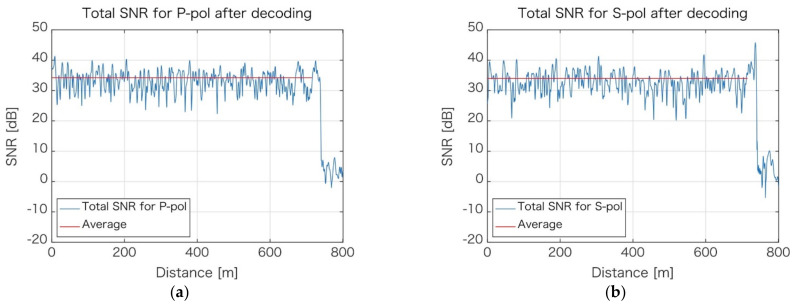
Signal-to-noise distribution along the fiber for P-polarization component (**a**) and S-polarization (**b**).

Figure 4 shows an example of the signal-to-noise ratio (SNR) distribution along the measured fiber. SNR is the ratio of the signal power to the noise power that corrupts the signal. In Figure 4, SNR is plotted separately, not only for each of the polarization components (Figure 4a,b), but also for individual processing frequency bands, denoted as P0, P1, P2, for P-polarization and S0, S1, and S2 for the S-polarization component. It can be observed that there are places along the fiber, which exhibit extremely low (negative) SNR values. If the datum from the individual band was only available, it would still be extremely challenging to obtain phase values at those phase fading locations. However, at any given location, if there is low SNR for one of the bands, then for the other bands—as they use other portions of the signal spectrum—SNR is high. Fortunately, the bands can (and are) combined, which means the fading phase location is virtually removed from the decoded signal. The SNR for combined bands for each of the polarization components are shown in Figure 5.

**Figure 6:**
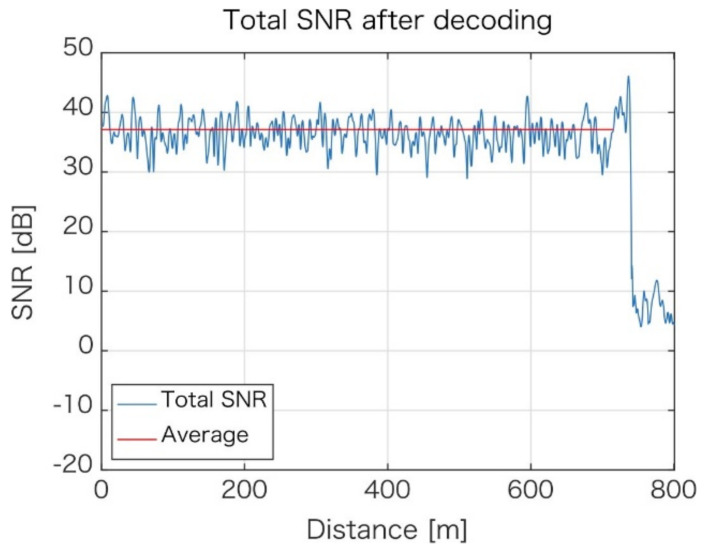
SNR distribution for the final (combined) data from all bands and polarization components.

To make the situation even better, the data for acquired polarization components are also combined to yield results, as shown in Figure 6. Along the entire length of the fiber, there is no location where phase fading could be observed, and the lower SNR is approximately +30 dB, with an average of +38 dB.

When combining the signal from individual components, in any measurement system, one should consider and determine the influence of the system noise contributing to (and influencing) the minimum measurable level, referred to as the inherent measurement system noise floor. This important aspect is discussed next.

### Noise Floor

For determining the noise floor of NBX-S4000, a typical procedure, in distributed optical fiber sensing and acoustic type of measurements, was used. First, the signal was acquired in a “non-acoustic” (also called “silent”) portion of the fiber. Next, the full spectral analysis of the signal performed, resulting in obtaining power spectrum density (PSD) of the signal, which in communication is also referred to as noise spectral density, or noise power density. The unit of PSD is power of noise over the frequency.

**Figure 7:**
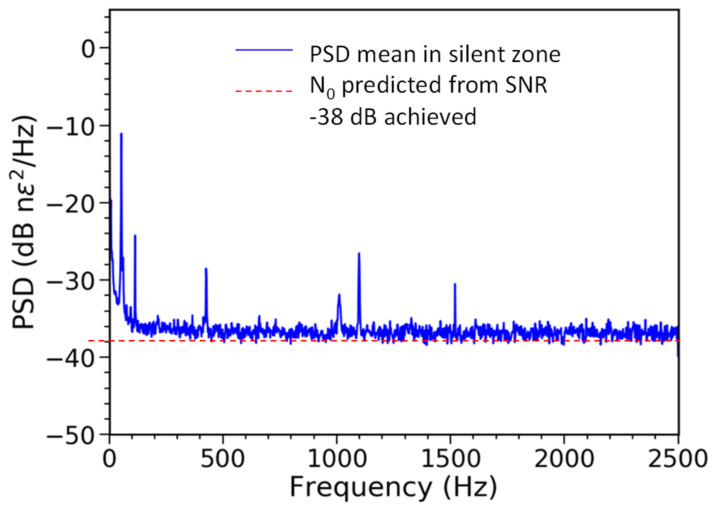
Noise power spectral density (PSD).

In Figure 7, as the very small strain value is detectable, nano-strain, nϵ, are used. Please note that, during the tests, we were not always able to create a fully soundproof nor vibration-free environment and, thus, some signals at the low frequency region below 100 Hz, and at some other specific frequencies, were detected. Those noise peaks were not present in data sets acquired in oil and gas wells, thus they can be ignored when discussing the noise floor and performance.

**Figure 8:**
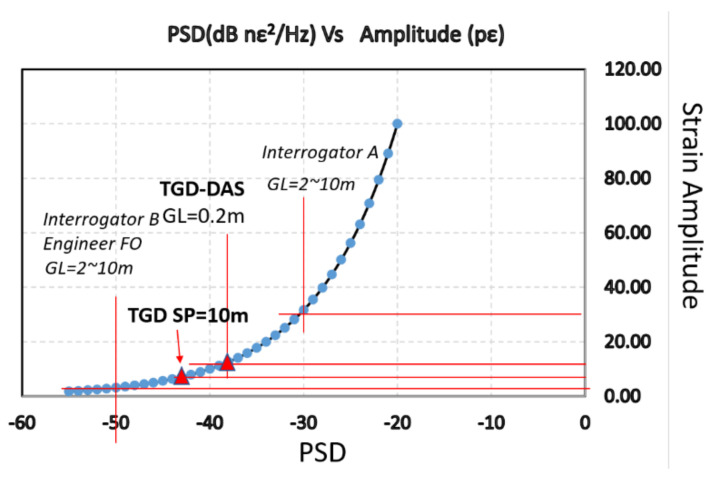
Performance of TGD-DAS in comparison with other reported results.

The noise floor of the instrument is at the level of N_0_ = −38 nϵ^2^ dB, with a 5 kS/s interrogation rate. This corresponds to √N_0_ = 0.0126 nϵ/√Hz. This means that a sustained input of sound waves of given frequency with an amplitude of 12.6 pϵ, or larger, can be detected. Figure 8 provides a comparison of the detectable signal, noise floor, and strain amplitude in reference to other interrogators, including the system, which uses engineered fiber to increase the SNR. In this figure, GL stands for gauge length.

We should also note that, unlike time-domain DAS methods where gauge length and spatial resolution are generally equal, in the OFDR type DAS, the gauge length and spatial resolution have much wider ranges of valid values and can be freely selected.

In the results presented in this paper, a 500 MS/s digitizer was used. This sampling rate provides the best (smallest) gauge length of 20 cm. Such a gauge length can extend the measurable, maximum strain change in a single time step to 5.4 µε; that is, two orders of magnitude higher than other available interrogators. This means the TGD-DAS can handle both extremes of the acoustic sensing in real-world applications, as it is capable of recording a strong vibration signal, and, at the same time, has the lowest noise floor in a standard SM fiber, and is able to record very weak signals.

Table 2 lists a few representative values for each variable. The actual performance is also linked to the interrogation rate, gauge length, and spatial resolution. Noting that the noise floor changes with these three quantities, this indicates that the design worked correctly. To the best of our knowledge, there is no other report that demonstrates this type of performance. The reasons for that might be signal-processing related, as usually papers and reports on DAS technology do not show the ‘raw’ interrogator performance, and include specialized processing steps before results are presented and passed to the user. Those additional processing steps might sometimes change the “engineering expectation”. In this paper, all of the results are obtained by using FPGA and show reliable performance at the industrial level.

One method to lower the noise floor in DAS measurements is by attempting to make Fiber Bragg Gratings (FBG) over the entire length of the optical fiber. This type of fiber is usually referred to as “engineered fiber”. The minimum noise floor that could be achieved with that special optical fiber was 3 pϵ [8]. However, as listed in Table 2, noise floor of −42 nϵ^2^ dB, corresponding to a detectable level of just 7.9 pϵ, can be obtained in standard SM with a gauge length of 3 m. This level is the highest ever achieved in the industry for standard SM fibers.

## 4. TGD-DAS Features, Advantages, and Future Improvements

In this section, we summarize the main advantages of TGD-DAS over other acoustic sensing methods and list improvements deployed in the NBX-S4000 interrogator.

### 4.1. Gauge Length and Spatial Resolution

Gauge length is defined as the interval length in the manufacturer’s method of analyzing the phase [6]. The spatial sampling interval of NBX-S4000 is set to 20 cm, due to requirement of the frequency band to restore the chirp signal. For this purpose, the gauge length can be arbitrarily set at intervals, with the step of 20 cm, to analyze the acoustic signal.

The spatial resolution of the TGD-DAS is approximately 1.8 m, which is inversely proportional to the gauge length as shown in Table 2, and is determined by the pulse width of the chirped pulses after compression. A minimum gauge length of 20 cm results in a maximum permissible strain as high as 5.4 µϵ, which is 14 times larger than the maximum permissible strain at a gauge length of 1.8 m, which is a gauge length equal to the spatial resolution, as used in typical implementations. This ability to handle large strains is significant in practical terms. Moreover, even if the strain exceeds the maximum permissible strain, the “wrapped” phase difference can still be measured, tracked, and unwrapped (in the time direction), allowing one to detect sound waves with even larger amplitudes. The details of the phase unwrapping procedure must meet specific mathematical conditions, and those depend on the sampling frequency and the frequency of the sound waves. As this topic is beyond the scope of this paper, the details are omitted here.

### 4.2. Resolution Change by Post-Processing

The distinct feature of the TGD-DAS method is its ability to change the spatial resolution of the already acquired data by simple post-processing. In the current implementation, resolution can be changed from 1.8 to 13.5 m, simply by selecting the appropriate number of bands, instead of using the default three. We demonstrate that feature in the following application examples, where the same measurement data can be converted to phase results with different spatial resolutions.

### 4.3. Future Improvements

After carefully analyzing measurement errors in the prototype interrogator, we concluded that the main source of those errors is the frequency drift of the light source. The drift exhibits in some abnormal signal components, as shown in Figure 7. One of the methods to remove this error, we believe, could be to establish a pre-processing method for first estimating and then removing the frequency shift of the light source using an acoustic reference fiber.

## 5. User Interface and Output Design of NBX-S4000

**Figure 9:**
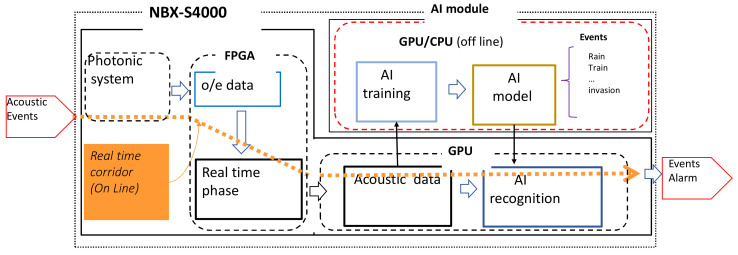
Events detection module, not an optic, but needed for AI processing.

The signal acquired by the interrogator unit can be automatically processed and used in events detection and a classification system. This requires a separate module for machine learning algorithm training. Figure 9 shows the overall scheme of automatic event detection as currently implemented in NBX-S4000.

An automatic monitoring system using acoustic signals in a wide range of applications and monitoring targets is an aspect of DAS that holds great promise. There are cases in which the AI module is executed in the acquisition mode (during signal acquisition) and some in the post-processing mode.

## 6. Application Examples

This section presents a series of application examples, in which a developed TGD-DAS acquisition method and interrogator unit not only demonstrated their capabilities and potential, but also were also thoroughly tested in the field environment. Results from seismic applications, measurements of submarine cables, hydraulic fracturing in oil and gas, as well as intrusion detection, are discussed. We conclude this section by presenting one of the distinct features of TGD-DAS, namely, a posteriori resolution changes for already acquired data.

### 6.1. Vertical Seismic Profiling (VSP) and Earthquakes Detection

First, we present two applications related to detecting seismic events.

#### 6.1.1. VSP

VSP is one of the most important techniques in geophysical exploration. It measures, using a set of sources and receivers, seismic wavefields to obtain properties of rock (such as velocity, impedance, attenuation, anisotropy). The seismic events are generated by the source, which is commonly a vibrator in onshore, and an air gun in offshore or marine environments. The receivers are usually placed along the depth (vertical axis) of the well.

There are many versions (configurations) of VSP type acquisitions, such as the zero-offset VSP, offset VSP, walkaway VSP, walk-above VSP, to name the most important ones. In the zero-offset VSP, the source is near the wellhead and above the vertical receiver array or sensing fiber. In the offset VSP, the source is located at some distance away from the vertical borehole. In the walk-away VSP, the source is moved in steps away from the well, while the receivers are held fixed. This approach effectively provides higher resolution seismic data and provides more continuous coverage than the standard offset VSP.

Until recently, geophones or accelerometers were used to record reflected seismic energy. With the increased sensitivity and accuracy of DAS, it became possible to obtain the VSP data using distributed sensing. This approach is preferable as it is economical (data are obtained faster and along the entire well) without need of moving the geophones array up and down the wellbore, and repeating the signal generation.

**Figure 10:**
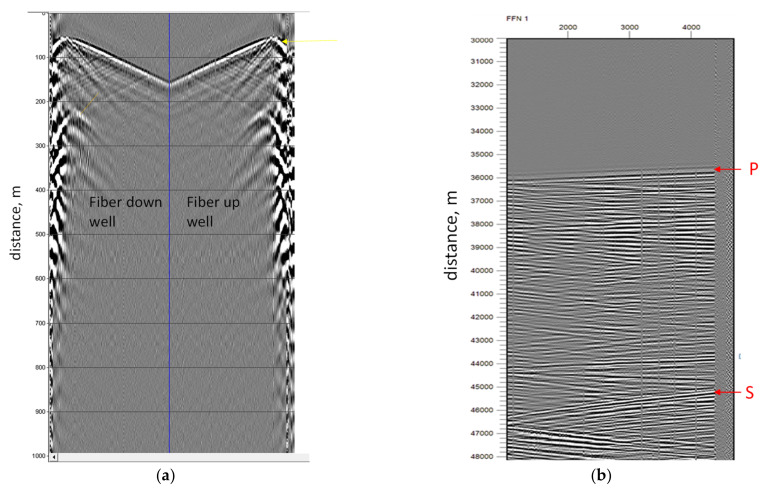
Seismic application examples. (**a**) Dataset acquired during walk-away VSP measurements in the vertical well; (**b**) seismic signal measured during the earthquake.

Figure 10a presents the results of measured data during walk-away VSP acquisition. The source was a small 15,000-pound P-wave vibrator, sweeping for 16 s over 10–150 Hz, linear. Four individual records were vertically stacked before cross-correlation and then cross-correlated with the pilot sweep to generate this record. The acquired data demonstrated the sensitivity and accuracy of TGD-DAS and the interrogator. The down-going and up-going reflected waves are clearly detected, providing data for further geological analysis. The obtained results were compared with geophones, confirming that the same level of accuracy was obtained, which means TGD-DAS can be used instead of geophones for VSP applications.

#### 6.1.2. Earthquake Detection

The second example of seismic data, acquired using the NBX-4000 instrument, deals with earthquake measurements (as presented in Figure 10b). This dataset shows the earthquake-generated waves. The signal was recorded during submarine cable measurements, off the Japan coast. The results clearly demonstrate the sensitivity of the instrument, as both P- and S-waves are recorded. Those waves are annotated in Figure 10b. Moreover, the line-separating signal before and after the earthquake can be clearly observed, which proves the high reliability of the acquired data and results.

### 6.2. Long Distance Range–Submarine Cables Measurements

Acoustic sensing in optical fibers is emerging as one of the most important technologies in submarine seismic monitoring applications [9,10,11]. The sole reason is the fact that existing submarine infrastructure can be converted into seismic and wave sensors by simply connecting the interrogator unit to many (already deployed) optical fibers in seabed cables. Until very recently, the main challenge and problem faced when trying to deploy was the limited distance, which DAS interrogator units could cover. The submarine infrastructure and cables are very long and can be accessed (easily) only from the coast. To verify that the NBX-S4000 interrogator is capable of measuring submarine cables without the need for any amplifiers, the test in the long-term deep sea floor observatory off Cape Muroto, Shikoku Island, Japan, was performed. The distance range to cover (measure) was 80 km.

The following acquisition settings were used:

- Temporal sampling rate was set to 0.5 kS/s;
- Spatial resolution set to (standard) 1.8 m;
- Gauge length was 0.2 m.

**Figure 11:**
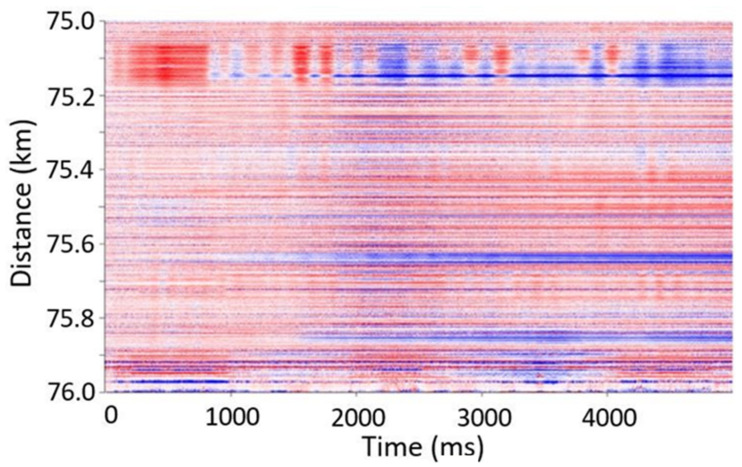
Observed acoustic signal in long-term deep sea floor observatory.

The sampling rate was selected to ensure that there is only one pulse travelling at any given time in the 80 km long fiber. The data were streamed to permanent storage on disks, selecting proper regions of interest. An example of acquired data with recorded ocean wave propagation is shown in Figure 11, in which a 1 km long section, between 75 and 76 km of the cable, was extracted and plotted. The signal-to-noise ratio of the acquired signal was still high and indicated that NBX-S4000 can be deployed for such marine applications. The full results of those tests, including detected seismic events at seabed, will be reported and published separately.

### 6.3. Hydraulic Fracturing and Fracture Hits Detection

Hydraulic fracturing, referred to as fracking, is a well stimulation process used in the oil and gas industry. It usually involves injecting water, sand, and chemicals under high pressure into a reservoir formation, of, generally, low-permeability rocks (e.g., shale). As a result, the fractures are created in the geological layer, and oil and/or gas can be extracted. The distributed sensing is particularly suited for monitoring of the fracking process, as it can provide real-time data, allowing engineering teams to ensure the correctness and state of the stimulation process, reached depths, and well integrity [8].

While measuring the signal on a fiber installed in well stimulation cannot be considered a great achievement, as the signal there is very strong, the detection of the signal on the neighboring well (referred to as a crosswell) certainly can. The ability to capture the signal there is of key interest for reservoir engineers, as it allows one to create the map of fracture networks and their azimuths, as well as to determine fracture geometry, spacing between them and far-field connectivity.

**Figure 12:**
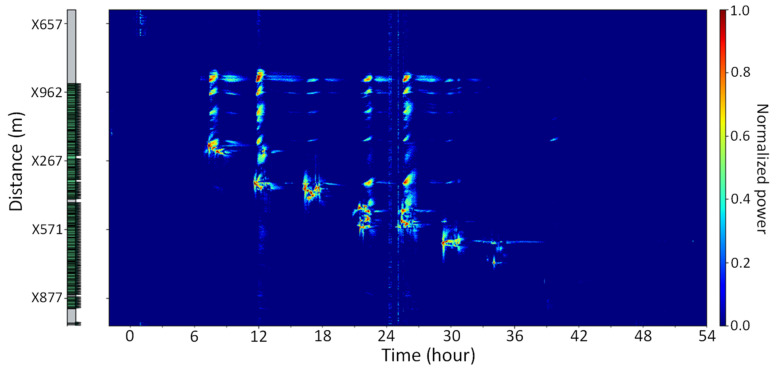
Signal detection (frac hits) on a crosswell.

The NBX-S400 instrument was already used in both types of monitoring, in the stimulated well and in the crosswell. We present, in Figure 12, the normalized power of the “frac hits” detected on the crosswell. It clearly demonstrates the quality of acquired data, which were additionally validated by comparing them with results obtained using the Rayleigh Frequency Shift (RFS) method [12]. This is another acquisition technique, developed by Neubrex, which finds its use in various monitoring applications, including the oil and gas industry, due to its capability to detect strain changes, high-resolution, and sensitivity.

### 6.4. Detection of Events Using “Dark Fibers”

The already built, extremely vast, telecommunication network of sub-surface fiber optics cables is commonly referred to as the “dark fiber” network [9]. While it serves its primary purpose by data transfer and communication, it can relatively (easily) convert into a sensing network, just by connecting it to the distributed sensing interrogator, which uses the available wavelength window and bandwidth. Such a sensing network can be used for earthquake detection and monitoring, structural health monitoring, or intrusion detection. In the latter, the system monitors the network for any malicious or unwanted events by performing continuous measurements. Generally, the detection system must also classify the signal; to do so, the signal quality must be high enough, not only to be detectable, but also to be distinguishable from other sources.

For intrusion detection on the railway tracks, a standard DAS instrument (which uses a single pulse technique) was previously used.

**Figure 13:**
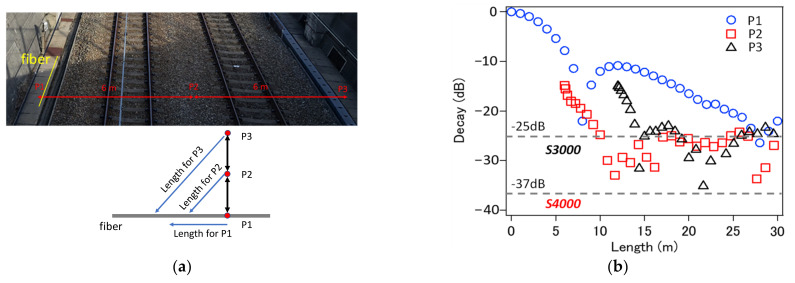
Intrusion detection example by NBX-S3000 (**a**) and (**b**) predicted improvement if NBX-S4000 is used.

Figure 13a presents the location of the sensing fiber and the distances to the key points along the tracks. The attenuations of the acoustic signal, generated by a person walking along the tracks, at indicated key points P1, P2, and P3 are plotted in Figure 13a. While the standard instrument used during the test could only detect intrusion at locations P1 and, partially only, P2 and P3. SNR and noise floor level of the NBX-S300 instrument, using a standard single-pulse technique, was far too high for reliable intrusion detection. In Figure 13b, the noise level presented in this paper, the TGD-DAS instrument was also indicated. With noise floor at −37 dB, the signal from even the furthest distance from the sensing fiber would be easily detectable. The tests using the NBX-S4000 interrogator will be repeated at the earliest opportunity, as it requires coordination with a railway company.

### 6.5. Extraction of Dispersion Curve from DAS Seismic Wave Measurements Data

In critical infrastructure, such as dams, knowing and monitoring the geotechnical properties of subsoil layers plays an essential role in ensuring its safety. Several different methods can be used to estimate such properties. While intrusive methods, such as drilling, can sometimes be used, the surface methods are preferable, as they are low-cost, noninvasive, and environmentally friendly. What is also important, in many cases, including dams, the surface methods do not destroy or leave any lasting marks on the surface of the test site.

In surface wave methods, waves are generated and used to measure propagation velocity profiles as a function of depth. In dam embankment, this velocity is related to the moisture content in it and can be used to monitor any potential subsurface leaks. As a signal source, vehicle driving on top of the bank is used [13].

There are several methods proposed to extract the dispersion curve. Among them, the phase-shift method is regarded as effective and robust, and is widely used. This method can be applied, not only toward analysis of the surface waves, but also body waves.

**Figure 14:**
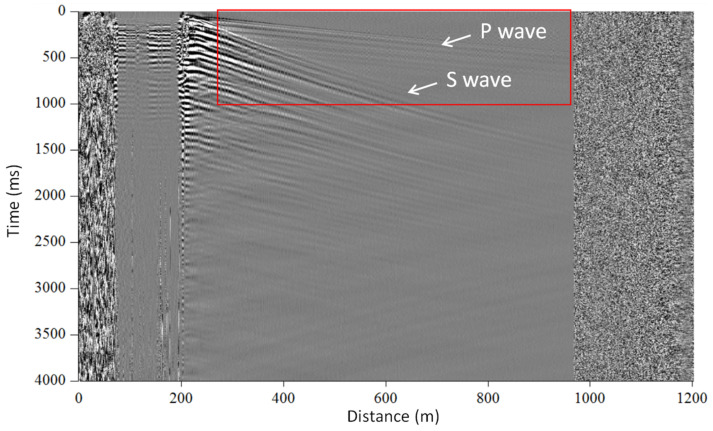
The measured seismic profiles of the surface wave.

The measured seismic profiles, including captured P- and S-waves, of the surface waves, are presented in Figure 14. The signal was obtained by stacking 35 datasets. The signal within the selected area (indicated by red rectangle) was then used as input data to the phase–shift dispersion curve analysis. The high quality of TGD-DAS (NBX-S4000) signal was compared directly with data obtained by geophones [14].

**Figure 15:**
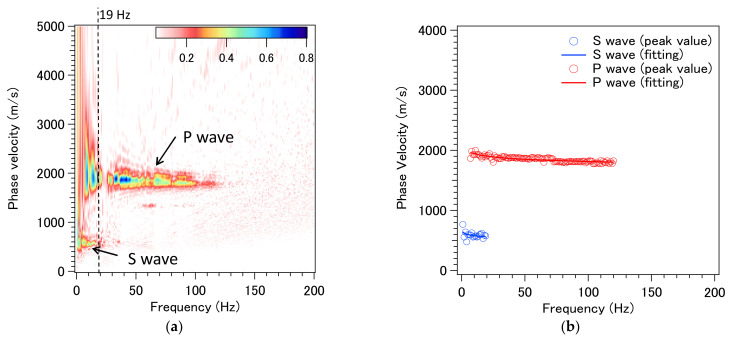
Velocity–frequency map (**a**) and dispersion curve (**b**).

First, during signal processing, the velocity-frequency map was generated (as shown in Figure 15a). The P-wave and S-wave are clearly seen in the figure. In the next step, peak values for each frequency line profile were determined, and the phase velocity of the P-wave and S-wave, as a function of frequency, are shown in Figure 15b.

We should note that, considering the very long lifetime of the optical cables and the high quality of the obtained signal and, therefore, the estimation results, it is possible to construct a permanent monitoring system using only sensing fiber and surface sources.

### 6.6. Variable Spatial Resolution for Already Acquired Data

Finally, we present one of the most distinct features of TGD-DAS, namely, its ability to modify the spatial resolution of the already acquired signal, simply by post-processing it. As described earlier in the paper, the spatial resolution in TGD-DAS depends on the selection of the frequency bands during the signal-decoding step. Hence, within limits determined by duration of the chirp, the spatial resolution can be changed. Currently supported are resolutions varying from 1.8 to 13.5 m.

**Figure 16:**
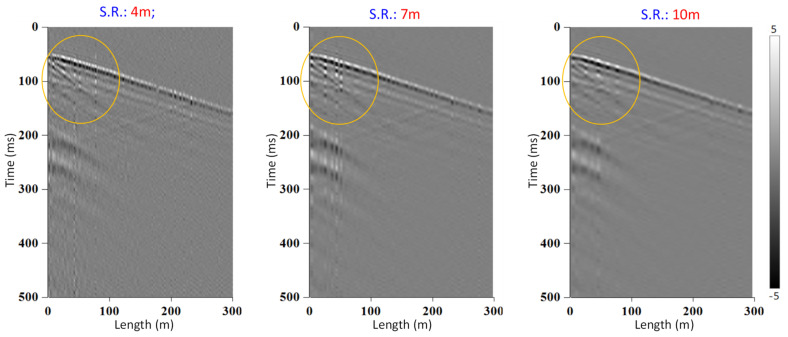
Results of signal processing with spatial resolution of 4, 7, and 10 m.

The need to change the spatial resolution in some applications arises from the fact that characteristics of wave propagation in different mediums are different. The most common use is seismology. Here we present an example of such a resolution change for the same VSP dataset. Figure 16 shows the results with applied resolutions of 4, 7, and 10 m.

**Figure 17:**
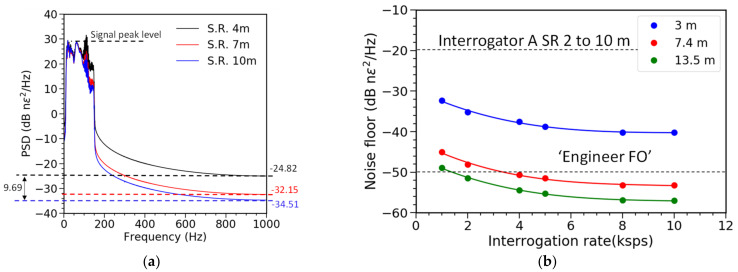
Spectral analysis results (**a**) with spatial resolution (SR) of 4, 7, and 10 m at interrogator rate of 2 kS/s (**b**) Performance for different interrogation rate.

The spectral analysis of the obtained signal, presented in Figure 17a,b, confirms that lower (longer) resolutions had also lower noise floor. In this case, the signal peak value is the same, as results for different resolutions are obtained from the same raw data, and the noise floor can be reduced by almost 10 dB.

Further analysis of the noise floor as a function of the interrogation rate and applied spatial resolution clearly demonstrates that performance of TGD-OFDR in standard SM fibers not only matches, but also surpasses that obtained by systems, depending on the specialty (weak FBG), engineered fibers. The deployment of engineered fibers has very practical implications, as it prevents from using the fiber for other sensing purposes and interrogators, so standard fibers are preferable.

Modifiable spatial resolution feature of TGD-DAS is particularly useful in any seismic application, as acquired data can be used to extract different features of the signal a posteriori, instead of determining them before any signal is measured.

## 7. Discussion

The DAS method and its implementation presented in this paper, compared to previous studies and applications, exhibit several substantial improvements and advantages. The most notable is that the synthesis of signals from extracted frequency bands, for both polarization components we detect, eliminates any phase fading from the acquired signal. Hence, the inherent problem in all OTDR-based systems, resulting in singularity when determining the phase value, is eliminated, simplifying the signal-processing part. Another substantial improvement is that spatial resolution is dependent on frequency range rather than pulse duration. This means that resolution can be modified after the signal is already acquired, and the resolution is independent of the measured fiber length. This feature makes TGD-DAS substantially different from any OTDR-based method. Results presented in the paper used varying resolutions between 1.8 and 13.5 m. We also presented in the field measurements, without any additional amplification, covering a distance of more than 80 km. In summary, the method, and its deployment in the NBX-S4000 interrogator, allows one to generate the signal with an optical power of a 200 m long pulse, but kept in a 2-m spatial resolution range.

## 8. Conclusions

This paper presents a practical DAS system that utilizes the advantages of both the frequency and time domain signal acquisition and processing. The TGD-DAS method, as well as the interrogator unit, achieves high precision, high spatial-resolution acoustic signal detection by frequency-division multiplexing, and real-time signal processing. The developed technique has room for further improvement, by expanding it to a wider frequency range and longer measurement distances. Those issues will be addressed in the future. The results obtained during the research presented in this paper revealed that performance and accuracy improvements are possible by developing a better light source and suppressing the noise caused by its varying frequency.

## Figures and Tables

**Table 1:** Typical application fields and technical requirements for DAS.

Application	Technical Requirements
Earthquake measurements, underwater wave survey.	Recording of waves for a few seconds, time and location accuracy are important.
Hydraulic fracturing (shale gas).	It is necessary to display the processed results on the screen every few seconds during several days of continuous operation. Low frequency is important.
Events detection and classification.	Expectations for an intelligent society through the introduction of AI technology.

**Table 2:** Noise floor as a function of gauge length.

Interrogator Rate kS/s	Gauge Length m	Spatial Resolution m	Noise Floor		Maximum Measurable Strain µε
			PSD dB nε^2^/Hz	Strain Amplitude pε	
2	0.2	1.8	−35	17.8	5.4
5	0.2	1.8	−38	12.6	5.4
5	3.0	3.0	−42	7.9	0.36
2	0.2	10.0	−45	5.6	5.4
